# A tool to address barriers in perinatal mental health screening, the PMH Connect: a perinatal mental health screening connection, education, and decision aid

**DOI:** 10.3389/fpsyt.2025.1699241

**Published:** 2026-02-02

**Authors:** Sara Wagner Moyer, Jaclyn D. Nunziato, Nicole W. Karjane, Alexis I. Rivera, Katherine L. Wisner, Amy L. Salisbury, Patricia A. Kinser

**Affiliations:** 1 Family and Community Health Nursing, Virginia Commonwealth University School of Nursing, Richmond, VA, United States; 2 Obstetrics and Gynecology and Health Systems Science and Interprofessional Practice Virginia Polytechnic Institute and State University Carilion School of Medicine, Roanoke, VA, United States; 3 Obstetrics and Gynecology, Virginia Commonwealth University School of Medicine, Richmond, VA, United States; 4 Bon Secours Mercy Health, Saint Francis Medical Center, Midlothian, VA, United States; 5 Developing Brain Institute, Children’s National Hospital, Washington, DC, United States

**Keywords:** perinatal mental health, mental health screening, patient provider communication, shared clinical decision making, patient information and communication

## Abstract

Mental disturbances and related symptoms in the perinatal period present a challenge to patients and providers alike, particularly regarding identification and appropriate management. Perinatal Mental Health (PMH) screening occurs in clinical settings on a more regular basis thanks to guidelines recommending the use of validated screening measures used at perinatal visits. However, patients report several concerns when completing these screeners and providers report barriers in addressing the results. To address barriers and enhance the PMH screening experience, our team of clinicians and researchers propose a tool – the PMH Connect: a Perinatal Mental Health Screening Connection, Education, and Decision Aid – to be given to the patient at the same time as a PMH screener. The PMH Connect provides brief anticipatory guidance about PMH symptoms, normalizing trauma-informed language about prevalence, and provides a connection to resources in a supportive, unobtrusive manner. PMH Connect helps patients feel heard and supported and provides resources before patients need them, which decreases the burden on patients and providers alike. Inspired by the Cycle to Respectful Care framework, PMH Connect is designed to shift power to patients themselves, as valued experts on their own care team, by offering them connections to information and resources through this simple tool. Our hope is that PMH Connect will bridge many of the barriers to effective PMH screening, assessment, and treatment by improving patients’ experiences and outcomes with the ultimate goal of optimizing screening effectiveness and care connection to improve maternal and infant health.

## Introduction

Perinatal mental health (PMH) describes emotional health and well-being during the full breadth of reproductive journey experiences (pre-conception, planning, pregnancy, postpartum, and parenting). Symptoms related to mental disturbances may first present or worsen during this time and Perinatal Mental Health (PMH) conditions such as depression, anxiety, and other psychiatric disturbances are among the most common obstetric complications and causes of maternal mortality (1). The American College of Obstetricians and Gynecologists (ACOG) recommends screening all patients for PMH symptoms with a validated tool at minimum once during pregnancy and again as a part of a comprehensive postpartum visit (2–4). Patients may have PMH screenings at multiple timepoints in both outpatient and inpatient settings throughout the continuum of perinatal care (e.g. routine prenatal visits, during inpatient care after birth, and during the comprehensive postpartum visit, during pediatric visits for their children) (2–4). However, patients and providers alike have expressed concerns about the environment of screening and appropriate responses to results of screening tools. In addition, patients with a history of traumatic experiences, racism/discrimination, and concerns about the stigma of PMH symptoms, may experience barriers preventing them from endorsing symptoms in screening tools; thus, their symptoms may remain under acknowledged and under-treated (5, 6). The aim of this perspective paper is to describe the development of the PMH Connect.

## Patient concerns about PMH screenings

It is our team’s clinical and research observation that pregnant and postpartum individuals describe significant concerns about the PMH screening process and experience. First, patients describe institutional level issues such as a failure to screen, barriers to screening (e.g., lack of access to appointments where screenings occur, disparities in screening practices), and lack of availability of appropriate follow-up care (e.g., limited or no availability or accessibility of specialized perinatal mental health providers and other resources, barriers to seeking care related to inability to attend appointments with children, paucity of childcare) (6–8). Second, patients describe interpersonal (provider) and procedural-level issues, including a lack of an individualized experience with PMH screenings (e.g., no anticipatory guidance provided about the purpose of the screening, or about the implications or outcomes of the screening) and a lack of follow-up or discussion by the provider (e.g., majority of providers and staff members do not discuss or review the screening form) (6–8). Third, at the internalized or personal-level, patients describe worry about stigma regarding PMH symptoms, misunderstandings about wording in screening tools, concerns about judgement or having negative consequences if they endorse symptoms, and reluctance to discuss their mental health with their provider. These factors result in patients selecting the “right answers” on the screener rather than communicating their experiences (8–12). Patients also report ambiguity in the screening tool items and/or that the tools do not adequately help them identify PMH symptoms (9–11, 13). Obstetric and primary care practices commonly ask patients to complete these screening tools on a clipboard or a computer-based patient portal while waiting for their provider as an efficient means to conduct such screening. The impersonal aspect of this approach may limit the utility of these screening tools, particularly in the most vulnerable patients. Some patients do overcome these barriers and use the form to communicate their experiences and endorse symptoms; however, many report that the form is never reviewed with them and they are never connected to care and support. In effect, the entire experience of completing a PMH screening may feel much more like “a box to check” or a risk-mitigation measure on the part of the healthcare provider (11).

In summary, the patient experience matters. The intent of PMH screening is to identify those experiencing symptoms, particularly those who might be at risk of PMH conditions, using validated screening tools to help create a patient-centered approach to follow-up and treatment (3, 4). Unfortunately, the current model lacks personalization and anticipatory guidance from the provider and is often confusing and stressful for patients. The negative stigma centered around mental health conditions is perpetuated by patients feeling dismissed, unheard, and therefore undertreated. The extant literature and anecdotal evidence make clear that pregnant and postpartum individuals feel unsure, unsupported, or confused while completing the screening; they feel disconnected from their providers when the screening is not reviewed or discussed; and, they feel dismissed when they honestly endorse symptoms but do not receive follow-up because they are not in the “very high risk” score category (7, 10–12). While a standardized approach to screening for PMH symptoms is a step forward, improving the screening experience and avoidance of undue distress and barriers to care for our patients is imperative.

## Question: what can be done to improve PMH screening?

### Answer: the PMH connect- a perinatal mental health screening connection, education, and decision aid

We propose that providers and patients alike could benefit from a simple, reproducible resource that accompanies PMH screening forms. The PMH Connect, a Perinatal Mental Health Screening Connection, Education, and Decision Aid, was developed by our team of researchers and clinicians based on extant literature in collaboration with feedback from stakeholders including patients, obstetricians, perinatal mental health providers, and interdisciplinary healthcare providers.

A strength of PMH Connect is that it is an inclusive educational decision aid that can serve to bridge gaps in communication that exist between provider and patient. The PMH Connect was intentionally developed as a multipurpose aid for use during PMH screening, serving as an information and education aid prior the patient completing the screening as well as a conversation guide and resource sheet after completing the screening. It strives to address the known systemic, procedural/interpersonal, and internalized/personal-level issues with PMH screening described above. It can be delivered in the form of a low-cost information sheet, either printed or via online methods, allowing it to seamlessly integrate into any method that a is currently used for patient communication. It uses trauma-informed language and integration of easily accessible technologies such as QR codes, which highlight the innovation in its simplicity and subsequently its potential for impactful change.

**Figure 1:**
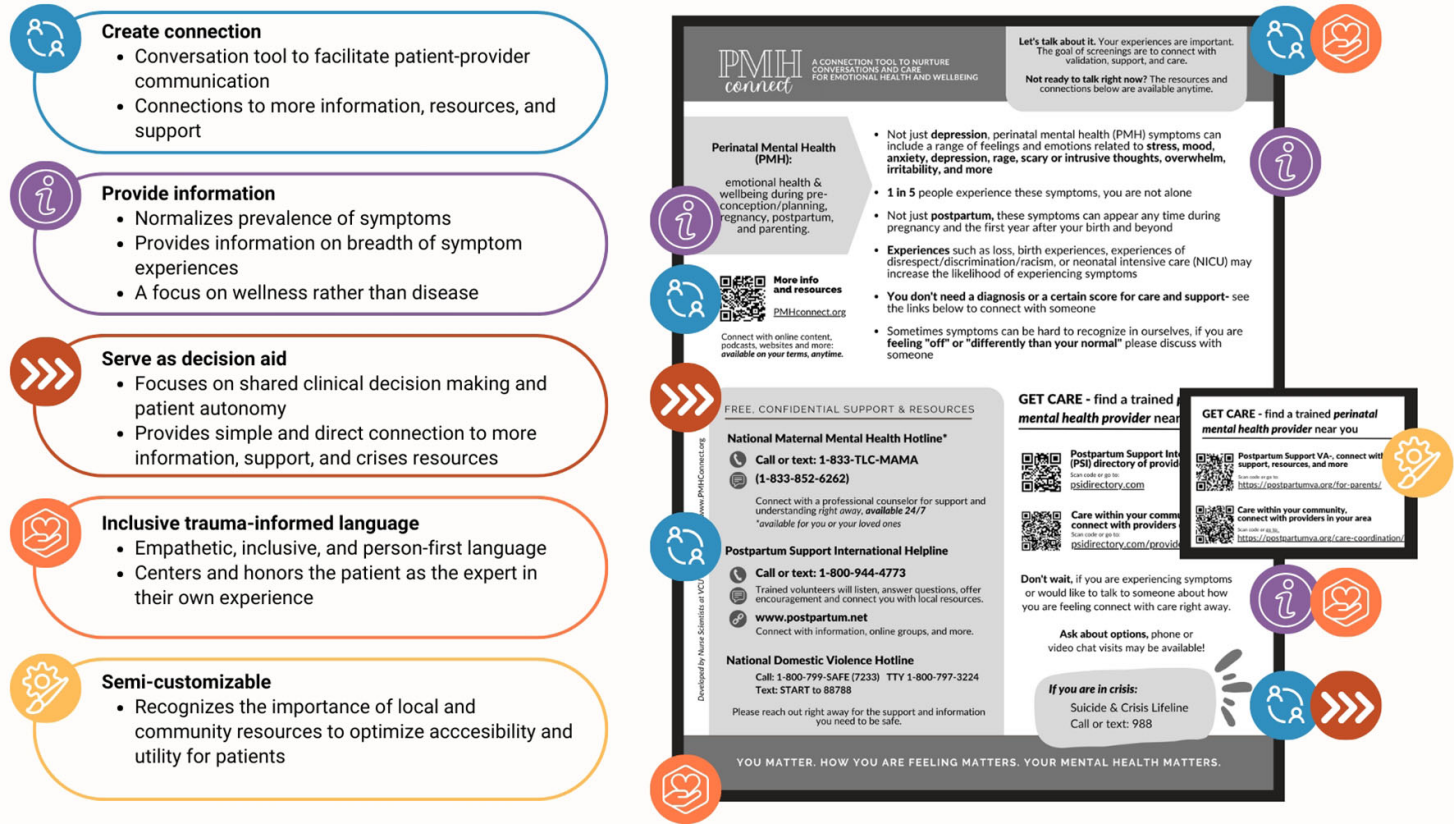
The main goals of the PMH Connect are to create connection, provide information, and serve as a decision aid and resource sheet. Semi-customized versions of the PMH Connect provide direct local (e.g., integrated behavioral health providers, local supports) or regional (e.g., state-based care coordination) resources.

As demonstrated in **Figure 1**, the goals of the PMH Connect are to:

#### Create connection

Some providers might feel that mental health conversations are beyond their scope. PMH Connect can be used to alleviate those concerns. As a conversation tool, the PMH Connect uses empathetic, inclusive, and person-first language that providers can emulate when explaining the purpose of PMH screening. PMH Connect facilitates connection of patients directly to care and additional information via scannable QR codes for easy access.

#### Provide information

As an educational tool, PMH Connect provides brief, simple educational points about PMH screening. With supportive language, PMH Connect provides anticipatory guidance and reviews PMH symptom prevalence and experiences. To do so, the tool: a) provides information on the full continuum of PMH symptoms and related experiences; b) uses an intentional trauma-informed approach incorporating inclusive person-first language to promote a focus on wellness rather than disease (14); and, c) communicates caring for the individual with language such as “You matter, how you are feeling matters, your mental health matters.”

#### Serve as a decision aid

PMH Connect is person-centered and empowers patients to connect with care and support for emotional health and wellbeing on their own terms via resources and QR codes linking to provider directories and/or to local services. As a decision aid, this tool respects patient autonomy and promotes shared clinical decision making. To do so, the tool: a) centers and honors the patient as the expert in their own experience; b) provides a simple connection avenue for the patient to connect with support and crisis resources; and, c) provides links for evidence-based websites for the patient to explore, including a direct link to Postpartum Support International’s main PMH provider directory.

#### Support the cycle to respectful care using inclusive trauma-informed language

The PMH Connect uses inclusive, trauma-informed language that aligns with core values of the Cycle to Respectful Care (15). In particular, the PMH Connect allows us as clinicians to: a) re-center ourselves on our professional pledge by facilitating an inclusive approach to PMH symptom screening; b) focus on holistic maternity care that acknowledges the breadth of experiences related to emotional health and well-being during the perinatal period; c) commit to the concept of respect and love for self and others by using person-first language relating to the PMH symptom experience; and, d) ensure we support birth equity and reproductive justice for populations experiencing health disparities by addressing and reducing barriers and harms experienced at internalized, interpersonal, and institutional levels during our current approach to PMH screenings (15).

## Background and frameworks for the development of PMH connect

### Three frameworks guided the development of the PMH connect

First, the Cycle to Respectful Care provides a framework for actionable changes to work toward respectful maternity care (15). This model charts a course for the development, dissemination, and implementation of the PMH Connect as an ongoing process promoting equity through a holistic approach to PMH screening and care that maintains our professional commitment to “do no harm.” (15) Underlying this model is the concept of cultural humility, in which we are called to engage in self-reflection and critique to enhance our openness in interactions with others (16). Through cultural humility, we strive toward a goal beyond “competence” with understanding individuals and cultures, to engage in constant openness to others’ unique experiences (16). The PMH Connect recognizes that the patient is the expert in their own experience and asks us, as clinicians, to constantly engage in curiosity about the patient in front of us. In engaging in cultural humility, our behaviors, attitudes, and policies will be most inclusive to the nuances of individuals’ experiences. Second, the Comprehensive Model of Mental Health during the Perinatal Period by Moyer & Kinser (2021) calls us to focus on key principles when conceptualizing PMH care and research: focused on wellness rather than disease; and recognition of the importance of inclusive, trauma-informed care experiences regarding PMH symptoms and overall well-being during the perinatal journey (14). Thirdly, a trauma-informed approach is important because of the high prevalence of individuals who have had some form of traumatic experiences during their lifetimes and because the potential sequelae of such experiences may be particularly relevant during the perinatal period; similarly, a trauma-informed model of care allows a focus on preventing new or recurrent traumas during the care experience. A trauma-informed model of care encourages providers to acknowledge the widespread impact of traumatic experiences, recognize signs and symptoms related to traumatic experiences and chronic stressors, implement responsive practices, and avoid re-traumatization; taking a “universal precautions” approach to integrating key principles of care for all patients, not just those with specifically identified trauma (17).

While the PMH Connect was developed based upon key literature and upon the frameworks highlighted above, it is relevant to note that the PMH Connect is also grounded in our personal experiences as researchers, as clinicians, and as mothers/parents. For example, the social worker on our team works regularly with individuals and families with Latinx backgrounds. It is her experience that pregnant/parenting individuals occasionally avoid deep discussions about mental health for a variety of reasons from stigma to language or educational barriers. When a resource or tool for discussion is made available, such as the PMH Connect, these individuals appreciate the opportunity to explore their feelings and to feel supported through their parenting journey. The clinicians and researchers on our team personally experience the disconnect when patients highlight that the wording of some PMH screening items is unclear, or when a patient reveals in candid conversation that they chose not to endorse an item because of concerns that endorsement triggers. As another example, one team member who is currently a parent of young children had personal experiences of “subthreshold” scores on PMH screeners that were never addressed by providers. Those of us in clinical settings have regularly had the positive feedback that our use of inclusive, trauma-informed language that recognizes the breadth of experiences of PMH related symptoms helped patients feel comfortable with the screening process and sharing their experiences.

### Best practices in PMH screening and future directions

As described in **Figure 1** and **Table 1**, the PMH Connect aims to address several gaps related to PMH screening.

**Table 1:** Recommendations about implementing best practices regarding PMH screening.

Best practices of PMH screening	Methods to implement best practices
Provide anticipatory guidance about PMH symptoms and screening	❖ Provide the PMH Connect alongside PMH screening form to patients ❖ Discuss the “why” for screening prior to having a patient complete the screening form → for example, “We care about your mental health, and this tool helps guide our conversations around overall well-being as well as additional support.”
Universal resourcing alongside conducting universal screening with validated tools*	❖ Provide the PMH Connect prior to screening, with every screening encounter regardless of scoring ❖ Screen every patient, regardless of perceived risk ❖ Use validated tools, but do not rely solely on these screening forms
Review results using nuanced, patient- centered conversations	❖ Use the PMH Connect as an information sheet and resource guide alongside a patient-centered assessment of mental health by discussing how current feelings and experiences compare to their norm Example of language that could be used: – “I see that your score was XXX on the PMH screening form. This corresponds to XX (no, mild, moderate, severe symptoms). How does that fit with your current experience?” – “Although the overall score on this screening is XX, I see that you marked that you are feeling XXX, could you tell me more about that?” – “How are things going? What stressors or pressures are you currently experiencing? What support might be helpful to you now?”
Engage in ongoing conversations, support, and care irrespective of PMH screening score or diagnosis throughout pregnancy, postpartum, and parenting	❖ Provide every person with the PMH Connect as an avenue for connection to resources when/if the individual decides to pursue them – the patient is an expert in their own care ❖ Use a spirit of cultural humility and constantly engage in conversations with individuals about their experiences, recognizing that personal, cultural, and systemic factors may affect how individuals perceive symptoms and choose to disclose their experiences to providers
Available on PMHConnect.org: - Versions in English & Spanish - Semi-customizable versions for clinicians to add contact information for local PMH providers	

In addition to the national resource, our team collaborates with clinical and community-based organizations, including state and local integrated care level teams, to create semi-customized versions of the PMH Connect on an on-going basis. As highlighted by the example in **Figure 1**. This approach recognizes the importance of local resources in addition national resources for maximum usefulness and accessibility for patients.

**Table 1**. Highlights recommendations for best practices for the use of PMH Connect along with mental health screenings; provide anticipatory guidance about the purpose of the screening, universal resourcing for every patient regardless of score or perceived risk, review the screening with a person-centered, nuanced discussion every time, and engage in ongoing conversations, care and support in a relational care setting whenever possible. The provision of this information sheet and decision aid to every patient who encounters a PMH screening every time, whether in person or online, should be one part of a multi-layered approach to optimizing PMH screening practices as a tool in our toolbox. However, the PMH Connect tool alone is by no means an exhaustive solution and importantly does not replace clinical assessment or in-depth discussion between the patient and their healthcare provider. Rather, this tool should be used as a universal approach to mitigate the aforementioned barriers and issues related to PMH screening in conjunction with person-centered clinical care. For example, this tool does not replace nuanced discussions between patients and providers reviewing the PMH screening results and addressing whether this aligns with the patient’s current experience.

Future directions should include methodological piloting of the PMH Connect with the continued collaboration of stakeholders at every level; patient/individual, healthcare provider/screener, as well as interdisciplinary health care system and payor level representatives, including assessments of acceptability and usability, incorporating user-feedback, and assessing barriers and facilitators of implementation. Immediate next steps and concurrent work by this team include assessing the perceived usefulness, acceptability, and appropriateness of the PMH Connect with varied stakeholders, using the Usefulness Scale for Patient Information Material (USE), Acceptability of Intervention Measure (AIM), Intervention Appropriateness Measure (IAM) scales alongside open-ended qualitative feedback.

Furthermore, we recognize that even with optimal and widespread implementation of the PMH Connect, additional systemic issues related to PMH screenings and care persist and are important to address. One such example, is the shortage of perinatal mental health trained providers, an issue which can be amplified by geolocation, such as in rural areas (18). Additionally, more work is needed to address systemic disparities affecting Black women and other historically marginalized and underserved groups through systemic and societal level change (5, 6, 18). Further, adapting PMH screening tools themselves to be fully understandable by diverse populations in the US and aligned with their symptom experiences is an important next step; one example is the EPDS-US, see more on this process in Moyer et al. (19, 20).

## Conclusion

Mental health and emotional well-being are essential to overall health. This is critically important in perinatal populations, where PMH conditions are top contributors to maternal mortality and transgenerational outcomes are impacted through interconnected maternal-child health. We recognize that incorporating PMH discussions and care to current healthcare visits can present a challenge to patients and providers alike. The PMH Connect is part of a layered approach to improving the patient and provider experience when navigating these issues, moving us toward a holistic approach to health and well-being. Although most obstetric care providers assess each patient’s mood and emotional well-being with a validated screening tool during the comprehensive postpartum visit, as recommended by the American College of Obstetricians and Gynecologists (1), time constraints and other obstacles encountered in clinical practice make it difficult for providers to perform these assessments in a meaningful way. Patients often report feeling uncomfortable with their providers, having trouble understanding the questions, or being concerned they may be judged if they endorse a current symptom or experience, these concerns are amplified for patients from historically marginalized groups and underserved communities. Therefore, the PMH Connect should accompany every episode of PMH screening during perinatal care, with the goal of providing anticipatory guidance and resources to patients before they need them, lessening the additional burden of seeking out these resources in a time of distress. PMH Connect, to be given to patients at the same time as the PMH screening tool, aims to help mitigate some of these challenges and provide context to patients regarding these assessments so they feel heard and supported.

Even in practices that optimally implement PMH screening tools, linking patients who screen positive for mental health disturbances to the appropriate resources can present an additional challenge. Availability and accessibility for appointments with mental health providers, especially those with perinatal specific training, are often limited, and patients struggle to get connected to support and treatment. The PMH Connect tool is designed to shift power to patients themselves, as valued experts on their own care team, by offering them connections to existing resources through this simple tool and linked online information and resources. Our hope is that the PMH Connect used in conjunction with best practices for PMH screening will bridge many of the barriers to effective PMH screening, assessment, and treatment by improving patients’ experiences and outcomes, with the ultimate goal of improving patient experiences, impacting disparities in maternity care and perinatal mental health screenings, and optimizing maternal and infant health outcomes.

The PMH Connect is freely available at www.PMHConnect.org

## Data Availability

The original contributions presented in the study are included in the article/supplementary material. Further inquiries can be directed to the corresponding author.
